# Influencing factors of health-related quality-of-life perceived by both children/adolescents patients with type-1 diabetes mellitus and their parents: A North-African study

**DOI:** 10.12688/f1000research.148074.1

**Published:** 2024-04-30

**Authors:** Imen Ben Abdesselem, Raoudha Kebeili, Khansa Derbel, Hichem Ben Said, Lamia Boughamoura, Jihene Bouguila, Helmi Ben Saad

**Affiliations:** 1 Department of Physiology and Functional Explorations, Hospital Farhat HACHED, Sousse, 4002, Tunisia; 2 Faculty of Medicine of Sousse, University of Sousse, Sousse, 4002, Tunisia; 3 Laboratory of Physiology, Faculty of Medicine of Sousse, University of Sousse, Sousse, 4002, Tunisia; 4 Research Laboratory "Heart failure" (LR12SP09), University of Sousse, Sousse, 4002, Tunisia; 5 Pediatrics, Hospital Farhat HACHED, Sousse, 4002, Tunisia

**Keywords:** Adolescent, Child, Diabetes mellitus type 1, Parents, HRQoL, North Africa

## Abstract

**Aim:**

To determine the influencing factors of health-related quality-of-life (HRQoL) perceived by North-African children/adolescents with type-1 diabetes-mellitus (T1DM) and their parents.

**Methods:**

It was a cross sectional study conducted in Tunisia. A total of 116 patients ( ***ie***; 43 children and 73 adolescents) and their parents were included. The Arabic validated version of the Pediatric Generic Core Quality-of-Life Inventory 4.0-Scale (PedsQL4.0) was used to evaluate the HRQoL of children/adolescents as perceived by patients and parents. The dependent data were the patients’ self-report and the parents’ proxy-report of the PedsQL4.0 total scores, and the independent data were the patients and parents characteristics.

**Results:**

Patients’ PedsQL4.0 total score was influenced significantly by siblings in the family > 2; lipodystrophy, and glycosylated hemoglobin (HbA1C). The combination of these factors explained 34.84% of the PedsQL4.0 total score variability. Parents’ PedsQL4.0 total score was influenced significantly by lipodystrophy, siblings; body mass index (BMI), hypoglycemia episodes, and HbA1C. The combination of these factors explained 56.92% of the PedsQL4.0 total score variability.

**Conclusion:**

T1DM patients self-reported HRQoL was influenced by siblings in the family > 2; lipodystrophy, and HbA1C. The parents’ proxy-report HRQoL was influenced by lipodystrophy, siblings, BMI, hypoglycemia episodes, and HbA1C.

**Abbreviations**
BMI: Body mass index
HbA _1_C: Glycosylated hemoglobin
HRQoL: Health related quality of life
PedsQL4.0: Pediatric Generic core Quality of life Inventory 4.0 Scale
PedsQL3.0: Pediatric Generic core Quality of life Inventory 3.0 Scale
PSI: Parents stress index
SD: Standard deviation
T1DM: Type1 diabetes mellitus

## Introduction

Type 1 diabetes mellitus (T1DM) is a major public health problem. ^1^ It is one of the most common chronic childhood diseases, with approximately 208000 children/adolescents worldwide affected in 2017. ^2^ The 2010-2020 decades have seen a significant increase in the incidence of T1DM worldwide. ^3^ This corresponds to an increase of more than 120000 new cases of T1DM per year in children/adolescents. ^2^ Between 2011 and 2021, and according to the 2022-report of the world health organization, the number of cases of T1DM in African children/adolescents increased fivefold ( ***ie***; from 4 to 20 cases per 1000 children/adolescents). ^4^ In low and middle- incomes countries, an increase of the incidence of T1DM was also observed. ^5^^–^^7^ T1DM influences negatively the physical and mental health, emotional development and vital prognosis of children/adolescents. ^2^^,^^5^^,^^8^ Traditionally, the management of T1DM has focused on achieving an optimal glycemic control in order to prevent long-term complications. ^6^ In addition to technological advances in the treatment of T1DM, more and more attention is being paid to the psychosocial component, and the family dynamics of the diabetic patient. ^9^ In fact, the complexity of the daily care plan, which continues throughout life, can affect negatively health-related quality of life (HRQoL) in children/adolescents with T1DM. ^2^^,^^6^^–^^16^ According to the Erikson's developmental theory, the disease brings significant changes to the development of the children/adolescents, and when the latter experience long-term treatment with painful injections, their normal mental development process is interrupted, and their self-esteem can be affected. ^17^ Every day, these children/adolescents live with the burden of this disease. ^17^ HRQoL, which is a concept, formed from the physical environment and a combination of social, emotional and school functions, ^11^^,^^16^^,^^18^^,^^19^ has become an important issue in the health field.

Generic and diabetes-specific HRQoL assessments are often advocated and have been shown to be useful in research. ^12^ For this reason, and in order to take decisions about the management of T1DM, approaches that focus on the triad ‘child-family-disease’ are needed. ^20^ Improving the HRQoL and the well-being of children/adolescents with T1DM is as important as metabolic control to prevent secondary morbidity. ^20^^–^^22^ Consequently, ‘modern’ diabetes care for children/adolescents has shifted from a purely medical approach to one that aims at optimal glycemic control and maximum HRQoL. ^21^ Despite the large number of international scientific studies examining HRQoL of diabetic children/adolescents, ^2^^,^^5^^–^^24^ only one Egyptian study were performed in North-Africa. ^5^ The Egyptian authors evaluated the HRQoL of 72 children with T1DM, tested how much it could be affected by their mood and family attitudes, and studied the relationship between these variables and the metabolic control of the patients. ^5^ They have included a control group of 72 children apparently healthy, non-diabetic, age and sex matched and siblings of the diabetic patients were included as a control group. ^5^ The authors have applied the Pediatric Generic core Quality of life Inventory 3.0 Scale (PedsQL3.0), parent stress index (PSI) questionnaire and glycosylated hemoglobin (HbA _1_C, %). ^5^ The authors reported ***i)*** significant positive fair correlations between the age, weight and body mass index (BMI) of diabetic children with the children and parent’ PedsQL; and ***ii)*** significant negative weak correlations between PSI score and children and parent’ PedsQL ( ***ie***; the higher parental stress, the lower HRQoL of the diabetic child (reported by both child and parent). ^5^

The results of the studies to examine the HRQoL of diabetic children/adolescents ^2^^,^^5^^,^^6^^,^^8^^,^^9^^,^^12^^,^^20^^,^^23^^,^^24^ can contribute to the evolution of professional practices and lead to the improvement of the delivery of health services to children/adolescents with T1DM in low- or middle- income countries, such as Tunisia. Therefore, the objectives of this study were to ***i)*** assess the HRQoL of Tunisian children/adolescents with T1DM as perceived by both patients (self-reported) and parents (proxy-reported); and ***ii)*** determine the influencing factors of the self-reported and proxy-reported HRQoL perceived by patients and their parents, respectively.

## Methods

### Study design

It was a cross-sectional study performed between January 2019 and February 2022 in the pediatric department of the Farhat HACHED Sousse university hospital, Tunisia. The study’s ethical approval was obtained from the ethics committee of the faculty of medicine of Sousse *(Reference: CEFMS 61/2021).* After obtaining their agreement, the parents of the children/adolescents signed a consent form. In addition, adolescents over 13 years old signed the consent form. The children/adolescents and their parents were informed about the ***i)*** study protocol and its aims, and ***ii)*** possibility of withdrawal from the research project at any time. During the period between June 2020 and February 2022, all recommended preventive measures to fight against the transmission of the severe acute respiratory syndrome coronavirus 2 were applied (eg; physical distancing of at least one meter, wearing a fitted facemask properly and cleaning hands frequently with alcohol-based hand rub or soap and water), During each study step, all recommended preventive measures to fight against COVID-19 transmission were applied. The study was conducted following the guidelines established by the STROBE statement. ^25^ In order to respect the anonymity and confidentiality of the data, a code was assigned to each patient.

### Population

**Figure 1.**
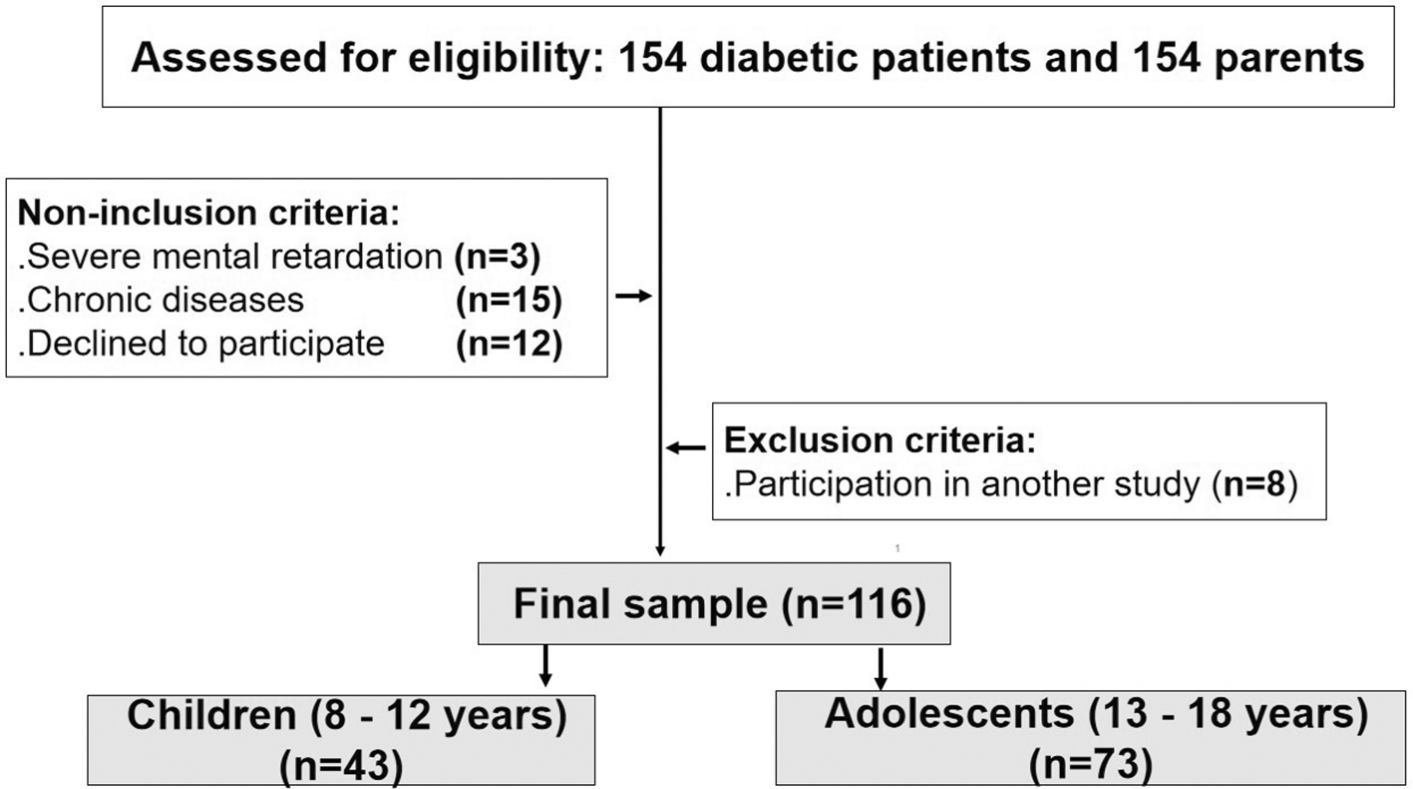
Study flow chart.

The source population was the children/adolescents (and their parents) attending pediatric outpatient consultations at the above cited hospital. The target population was the children/adolescents with T1DM who were consulted during the study period. Only children/adolescents aged 8 to 18 years, who were accompanied by their parents, who had a medical diagnosis of T1DM at least one year before the inclusion in the study, and who received only insulin analogues for at least three months were included in the study. Children/adolescents who had a mental retardation, a sensor neural disorder that interferes with normal communication; or another chronic disease, such as celiac disease, were not included in this study. Children/adolescents who participated in another experimental survey during the study period were excluded from the statistical analysis. Figure 1 exposes the study flow chart.

### Sample size

The sample size was appraised according to the following formula ^26^: *N* = ( *Z*_*α*_) ^2^ *s* ^2^/ *d* ^2^, where “ ***s***” is the standard deviation (SD= 9.79) and “ ***d***” is the accuracy of estimate or how close it is to the true mean (= 76.51). Given the pioneering nature of this study, the above “ ***s***” and “ ***d***” data were collected from a previous work including 503 adolescents with T1DM, ^27^ aiming to evaluate the HRQoL via the diabetes quality of life instrument for youth. ^28^ In the aforementioned study, the percentage mean score of the total HRQoL for all adolescents was 76.51±9.79. “Z _α_” is the normal deviate for a one-tailed alternative hypothesis at a level of significance (Z _α_ equal to 1.28 at an error rate of 0.10%). The appraised sample size gives a sample of 116 patients. The assumption of 25% for the non-inclusion and exclusion criteria gives a revised sample of 154 patients (154 =116/ (1.0-0.25)).

### Data collection and procedure

One investigator ( *IBA* in the authors’ list) was in charge of administering and explaining the different components of the questionnaire to children/adolescents and their parents. On the beginning of the consultation day, the questionnaire was given to patients and parents independently in order to improve the response rate. The questionnaire was then collected at the end of the morning of the same day. The questionnaire included two parts and the time taken to complete it was 15 minutes.

The first part of the questionnaire, which was written in Arabic, was pretested, structured, and self-administered by the researcher ( *IBA* in the authors’ list). It was related to personal data of both diabetic patients and their parents. For parents, the following data were collected: accompanying tutor ( ***ie***; mother; father), marital status ( ***ie***; married; divorced), schooling-level ( ***ie***; illiterate or primary or secondary school; university), and socio-economic level [ ***ie***; low ( ***eg***; unskilled worker, jobless); high ( ***eg***; skilled worker, farmer, manager)]. For patients, the following data were collected: sex ( ***ie***; male; female), age ( ***ie***; years, children (8 to 12 years), adolescents (13 to 18 years)); BMI (kg/m ^2^), corpulence status ( ***ie***; normal weight; overweight or obese), ^29^ siblings in the family ( ***ie***; number sibling > 2), schooling-level [ ***ie***; low (primary or preparatory school) and high (secondary school)], schooling-performance ( ***ie***; normal; repeating or stop); family history of diabetes mellitus ( ***ie***; yes, no), onset age and seniority of T1DM ( ***ie***; year), person in charge of insulin injection ( ***ie***; patient, parents), dietetic education ( ***ie***; yes, no), diet ( ***ie***; yes, no), hypoglycemia episodes during the last three months before the inclusion in the study ( ***ie***; number, yes, no), average glycaemia during the hypoglycemia episodes ( ***ie***; g/l), home self-monitoring ( ***ie***; yes, no), home self-monitoring frequency ( ***ie***; never, > 1), most recent HbA _1_C level ( ***ie***; %), duration of HbA _1_C control ( ***ie***; control < 3 months, control > 6 months), lipodystrophy ( ***ie***; yes, no), previous hospitalization ( ***ie***; yes, no), and etiologies of hypoglycemia ( ***ie***; lipodystrophy, diet, stop insulin). Two subgroups of patients ( ***ie***; non-active; active) were formed according to regular sports activity based on the response to the following question ^30^: do you practice any sports activities outside of school? Some data ( ***eg***; family history of T1DM, HbA _1_C levels and potential previous hospitalizations) were extracted from the patients’ medical records.

The second part of the questionnaire was reserved to the PedsQL4.0. ^10^^,^^31^ The latter was developed to assess the HRQoL of children/adolescents (self-reported) as well as their parents’ (proxy-report). ^10^^,^^31^ HRQoL is assessed in four domains: physical function *(8 items)*, emotional function *(5 items),* social function *(5 items),* and school function *(5 items).* ^10^^,^^31^ The following three scores are determined: total score, physical health score, and psychosocial health score (covering emotional, social and academic function). ^10^^,^^31^ Total scores are obtained by adding the scores and dividing them by the total number of items completed. Patients and parents were asked to rate the ‘problem in the past three months’ on 5 Likert scales from 0 to 4 ( ***0*** = never a problem; ***1*** = almost never a problem; ***2*** = sometimes a problem; ***3*** = often a problem, ***4*** = almost always a problem). Then each item was scored backwards and linearly transformed on a scale of 0 to 100 ( ***0*** = 100, ***1*** = 75, ***2*** =50, ***3*** = 25, ***4*** = 0), so that the highest score indicates better HRQoL. ^10^^,^^31^ The PedsQL4.0 has appropriate forms for child self-assessments and parent proxy reports, ^31^ and has shown good internal consistency, reliability and validity. ^10^ The validated Arabic version of the PedsQL4.0 was applied. ^15^ Permission to use the PedsQL 4.0 was obtained from the authors.

### Statistical analysis

**Data expression**. The Kolmogorov Smirnov test was used to analyze quantitative data’s distribution. All quantitative data have a normal distribution, and therefore were expressed as means ± standard deviation. Categorical data were expressed as number (frequency).

**Comparisons parents vs. patients**. Comparisons of the PedsQL4.0 scores between parents and patients were performed by the Student-T test.

**Univariate and multiple regression analysis (influencing factors)**. The dependent variable ( ***ie***; PedsQL4.0 total scores of patients and parents) was normally distributed. Student-T test were used to evaluate the associations between the PedsQL4.0 total score and categorical data [ ***ie***, parents’ data (accompanying tutor, marital status, schooling-level, and socio-economic-level); patients’ data (sex, age range, siblings in the family, schooling-level, schooling-performance, corpulence status, charge of injections, dietetic education, diet, regular physical activity, hypoglycemia, home self-monitoring, frequency of home self-monitoring, duration of the control of HbA _1_C, lipodystrophy, previous hospitalizations, and family history of diabetes mellitus)]. The Pearson product-moment correlation coefficients (r) evaluated the associations between the PedsQL4.0 total score and continuous data [ ***ie***; age, siblings, BMI, hypoglycemia episodes, average glycaemia during hypoglycemia episodes, HbA _1_C, onset age of T1DM, and seniority of T1DM]. The linearity of the association between the PedsQL4.0 total scores and the continuous data was checked graphically by plotting each regressor against the PedsQL4.0 total scores. Only significantly and linearly associated data were entered into the model.

Linear regression models were used to evaluate the independent data explaining the variance in PedsQL4.0 total scores. Candidate data were put into the model with a stepwise selection method. To determine entry and removal from the model, significant levels of 0.15 and 0.05 were used, respectively. No colinearity between predictors was detected with variance inflation factors. The linear regression models were evaluated by the (r) and the standard error. All statistical procedures were performed using a statistical software (StatSoft, Inc. (2014). STATISTICA (data analysis software system), version 12. www.statsoft.com, RRID: SCR_014213). The significance level was set at p<0.05.

## Results

Among the 154 patients assessed for eligibility, and after applying the inclusion, non-inclusion, and exclusion criteria, 116 diabetic patients aged 8 to 18 years and 116 parents were included in the study ( Figure 1).

### Parents and patients characteristics

Table 1 illustrates the parents’ and patients characteristics, respectively. Its main conclusions were: ***i)*** almost 92% of parents have a low socio-economic-level; ***ii)*** there was a predominance of adolescents and females (63% and 64%, respectively), ***iii)*** 56% of patients have normal weight, ***iv)*** the mean of siblings was 3±1, and 71% of patients had more than 2 siblings in the family, ***v)*** the means of hypoglycemia episodes and HbA _1_C were 4.3±1.5, and 10±1, respectively, and ***vi)*** 69% of patients have lipodystrophy.

**Table 1.** Characteristics of parents and patients with type-1 diabetes mellitus (T1DM) (n=116).

**PARENTS**		
**Category**		
**Accompanying tutor** ^**b**^	Mother	87 (75)
**Marital status** ^**b**^	Married	114 (98)
**Schooling-level** ^**b**^	Illiterate or primary or secondary school	91 (78)
	University	25 (22)
**Socio-economic-level** ^**b**^	Low	92 (79)
**PATIENTS**		
**Anthropometric and socio-demographic data**		
**Age** ^**a**^	Yrs	14±4
**Age range** ^**b**^	Adolescents (13-18 yrs)	73 (63)
**Sex** ^**b**^	Female	74 (64)
**Body mass index** ^**a**^	kg/m ^2^	22.3±3.5
**Corpulence status** ^**b**^	Normal weight	65 (56)
	Overweight or obese	51 (44)
**Siblings** ^**a**^	Number	3±1
**Siblings in the family** ^**b**^	> 2	82 (71)
**Schooling-level** ^**b**^	Low	81 (69)
	High	35 (30)
**Schooling-performance** ^**b**^	Normal	68 (59)
	Repeating or stop	48 (41)
**Clinical and biological data (last 3 months)**		
**Charge of injections** ^**b**^	Yes	65 (56)
**Dietetic education** ^**b**^	No	79 (68)
**Diet** ^**b**^	No	107 (93)
**Regular physical activity** ^**b**^	Active	88 (76)
**Home self-monitoring** ^**b**^	Yes	61 (53)
**Frequency of home self-monitoring** ^**b**^	Never	51 (44)
**Hypoglycemia** ^**b**^	Yes	114 (98)
**Hypoglycemia episodes** ^**a**^	Number	4.3±1.5
**Average glycaemia during hypoglycemia episodes** ^**a**^	g/l	0.53±0.12
**Lipodystrophy** ^**b**^	Yes	80 (69)
**Glycosylated hemoglobin (HbA**_**1**_ **c)** ^**a**^	(%)	10±1
**Duration of the control of HbA1c** ^**b**^	<3 months	110 (95)
**Previous hospitalization** ^**b**^	Yes	47 (41)
**Onset age of T1DM** ^**a**^	Yrs	7±3
**Seniority of T1DM** ^**a**^	Yrs	7±4
**Family history of diabetes mellitus** ^**b**^	Yes	32 (28)
**Etiologies of hypoglycemia** ^**b**^	Lipodystrophy	71 (61)
	Diet	113 (97)
	Stop insulin	3 (3)

Data were

^a^ Mean±standard deviation.

^b^ Number (%).

### Comparison of patients’ self-report and parents’ proxy-report of PedsQL4.0 scores

Patients and parents had comparable scores of physical health (67±20 vs. 70±21, p=0.271, respectively), emotional function (53±20 vs. 57±21, p=0.074, respectively), social function (81±18 vs. 83±18, p=0.502, respectively), academic function (70±21 vs. 70±21, p=0.886, respectively), and total score (68±13 vs. 70±14, p=0.180, respectively).

### Univariate analysis between PedsQL4.0 total scores of patients and parents, and categorical and quantitative data

Tables 2 and 3 expose the univariate analysis between PedsQL4.0 total scores of patients and parents and categorical and quantitative data, respectively. Among patients, the following four data influence the PedsQL4.0 total score: siblings in the family, dietetic education, lipodystrophy, and HbA _1_C. In parents, the following eight data influence the PedsQL4.0 total score: siblings in the family, corpulence status, dietetic education, lipodystrophy, siblings, BMI, hypoglycemia episodes, and HbA _1_C.

**Table 2.** Univariate analysis between the pediatric generic core quality of life inventory 4.0 total score (patients’ self-report and parents’ proxy report) and demographic and clinical characteristics.

Data	Category	Patients’ self-report	P-value	Parents’ proxy-report	P-value
**Patients (n=116)**					
**Sex**	**Male** (n=42)	70±10	0.109	73±9	0.061
	**Female** (n=74)	66±14		68±16	
**Age range**	**Children** (n=43)	68±11	0.824	70±16	0.887
	**Adolescents** (n=73)	68±14		70±12	
**Siblings in the family**	**≤ 2** (n=82)	66±13	**0.005** *	68±15	**0.005** *
	**> 2** (n=34)	73±11		76±9	
**Schooling-level**	**High** (n=67)	68±12	0.689	71±14	0.309
	**Low** (n=49)	67±15		69±14	
**Schooling-performance**	**Normal** (n=68)				
	**Repeating or stop** (n=48)				
**Corpulence status**	**Normal** (n=65)	67±13	0.669	68±15	**0.046** *
	**Overweight or obese** (n=51)	68±3		73±12	
**Charge of injections**	**Patient** (n=65)	70±12	0.110	72±14	0.117
	**Parents** (n=51)	66±13		68±14	
**Dietetic education**	**Yes** (n=37)	72±12	**0.025** *	75±11	**0.016** *
	**No** (n=79)	66±13		68±15	
**Diet**	**Yes** (n=9)	74±13	0.131	78±8	0.087
	**No** (n=107)	67±13		69±14	
**Regular physical activity**	**Active** (n=88)	67±13	0.440	69±14	0.233
	**Inactive** (n=28)	69±13		73±15	
**Hypoglycemia**	**Yes** (n=114)	67±13	0.150	70±14	0.095
	**No** (n=2)	89±2		86±5	
**Home self-monitoring**	**Yes** (n=61)	67±11	0.789	69±4	0.429
	**No** (n=55)	68±15		71±13	
**Frequency of home self-monitoring**	**Never** (n=51)	69±15	0.514	72±12	0.169
	**≥ 1** (n=65)	67±11		69±15	
**Duration of the control of glycosylated hemoglobin**	**<3 months** (n=110)	68±13	0.787	70±14	0.366
	**3-6 months** (n=6)	69±15		75±7	
**Lipodystrophy**	**Yes** (n=80)	66±13	**0.024** *	77±11	**0.001** *
	**No** (n=36)	72±12		67±14	
**Previous hospitalizations**	**Yes** (n=47)	66±13	0.903	71±13	0.332
	**No** (n=69)	68±13		69±15	
**Family history of diabetes mellitus**	**Yes** (n=32)	67±16	0.617	68±15	0.253
	**No** (n=84)	68±12		71±13	
**Parents (n=116)**					
**Accompanying tutor**	**Mother** (n=87)	67±14	0.721	69±14	0.165
	**Father** (n=29)	68±11		73±14	
**Marital status**	**Divorced** (n=2)	77±10	0.297	79±3	0.367
	**Married** (n=114)	68±13		70±14	
**Schooling-level**	**Preparatory school** (n=91)	67±13	0.5731	70±14	0.391
	**University** (n=25)	69±11		72±3	
**Socio-economic-level**	**Low** (n=92)	67±14	0.243	69±14	0.252
	**High** (n=24)	69±10		73±13	

Data were median±standard deviation.

^*^ P-value (Student T test) < 0.05: comparison between 2 categories for the same group (patients or parents).

**Table 3.** Univariate analysis between the pediatric generic core quality of life inventory 4.0 total score (patients’ self-report; parents’ proxy report) and quantitative data.

Patients data	Unit	Patients’ self-report (n=116)		Parents’ proxy report (n=116)	
		r	p-value	r	p-value
**Age**	Yrs	0.0331	0.724	0.0530	0.571
**Siblings**	Number	-0.1774	0.0567	-0.3415	**0.001** *
**Body mass index**	kg/m ^2^	0.1815	0.051	0.3163	**0.001** *
**Hypoglycemia episodes**	Number	-0.0610	0.515	-0.2357	**0.011** *
**Average glycaemia during hypoglycemia episodes**	g/l	0.0221	0.814	-0.1071	0.252
**Glycosylated haemoglobin**	%	-0.1946	**0.036** *	-0.2647	**0.004** *
**Onset age of diabetes mellitus**	Yrs	-0.0497	0.596	-0.1180	0.207
**Seniority of diabetes mellitus**	Yrs	0.0966	0.302	0.1346	0.149

**r**: correlation coefficient.

^*^ p-value < 0.05.

### Multivariate linear regression analysis: predictors of the PedsQL4.0 total scores of patients and parents

Table 4 exposes the multivariate linear regression analysis. Among patients, only three data influence the PedsQL4.0 total score: siblings in the family, lipodystrophy and HbA _1_C. Altogether, they explain 34.84% of the PedsQL4.0 total score variance. In parents, only five data influence the PedsQL4.0 total score: lipodystrophy, siblings, BMI, hypoglycemia episodes, and HbA _1_C. Altogether, they explain 56.92% of the PedsQL4.0 total score variance.

**Table 4.** Multivariate linear regression analysis: predictors of the pediatric generic core quality of life inventory 4.0 total score (PedsQL4.0) of patients and parents.

Independent variables	Category/unit	B	Standard error of B	Cumulative correlation coefficient
**Patient self-report (n=116)**				
**Constant**	-	88.10	-	-
**Siblings in the family >2**	Yes=1, No=0	- 6.84	2.1521	0.2575
**Lipodystrophy**	Yes=1, No=0	- 4.29	2.6577	0.3225
**Glycosylated hemoglobin (HbA**_**1**_ **c)**	%	- 1.20	8.3276	0.3484
**Parents proxy-report (n=116)**				
**Constant**	-	80.77	-	-
**Lipodystrophy**	Yes=1, No=0	- 6.97	2.1865	0.3419
**Siblings**	Number	- 3.73	4.0199	0.4641
**Body mass index (BMI)**	kg/m ^2^	1.03	8.5660	0.5265
**Hypoglycemia episodes**	Number	- 1.69	8.6265	0.5610
**HbA**_**1**_ **c**	%	- 0.96	11.4378	0.5692

**B:** non-standardized regression coefficient.

**PedsQL4.0 of patients =** 88.10-6.84 × Siblings in the family > 2-4.29 × Lipodystrophy - 1.20 × HbA _1_c.

**PedsQl4.0 of parents =** 80.77-6.97 × Lipodystrophy - 3.73 × Siblings + 1.03 × BMI -1.69 × Hypoglycemia episodes - 0.96 × HbA _1_c.

## Discussion

The main results of the present study including 116 diabetic children with T1DM (and their parents) were that the diabetic patients self-reported HRQoL was influenced by siblings in the family >2; lipodystrophy, and HbA _1_C, and that the parents’ proxy-report HRQoL was influenced by lipodystrophy, siblings; BMI, hypoglycemia episodes, and HbA1C.

Patients living with T1DM experience various challenges, related mainly to a restrictive lifestyle, multiple daily insulin injections, and monitoring of blood glucose levels. ^6^ The process to manage this chronic disease impacts the HRQoL of the children/adolescents, and interferes also with the familial dynamics and the parents’ HRQoL. ^32^ This study provides results useful for assessing HRQoL in patients with chronic diseases living in Tunisia, and North Africa. To the best of the authors’ knowledge, this is the first North-African study that evaluates the influencing factors of the HRQoL of children/adolescents with T1DM as perceived by patients and parents.

### Tunisian diabetic HRQoL scores

The Arabic version of PedsQL4.0 is understandable and usable in Tunisia. ^15^ It has a high validity and reliability. ^15^ Table 5 exposes the PedsQL4.0 scores among different populations of diabetic children/adolescents with TIDM. ^2^^,^^6^^,^^8^^,^^10^^–^^16^

**Table 5.** Pediatric generic core quality of life inventory 4.0 data (scores and influencing factors) among different populations of diabetic children/adolescents with type 1 diabetes mellitus.

	This study	USA	Greece	USA	Iran	Greece	Kuwait	Kuwait	Turkey	Ethiopia	Ethiopia
**Reference**		^10^	^11^	^12^	^13^	^14^	^15^	^16^	^2^	^6^	^8^
**Number**	116 pat A par of each child	300 pat 308 par	89 pat 89 par	122 pat A par of each child 390 CG	94 pat A par of each child	117 pat 128 control group Par in 2 groups	112 pat.131 par 104 CG	436 pat 389 CG Par in 2 groups	149 pat A par of each child	470 pat A par of each child	379 pat
**Age (yrs)**	8-18 ^**a**^	5-18 ^**a**^	2-18 ^**a**^	11.5 ^**b**^	8-18 ^**a**^	5-18 ^**a**^	2-18 ^**a**^	2-18 ^**a**^	8-18 ^**a**^	8-18 ^**a**^	11.65 ^**b**^
**Physical health**	67±20 ^**c**^	86±13 ^**c**^	87±12 ^**c**^	85±13 ^**c**^	69±17 ^**c**^	80±14	80±11 ^**c**^	75±12 ^**c**^	82±15 ^**c**^	82±20 ^**c**^	89±14 ^**c**^
**Emotional function**	53±20 ^**c**^	72±20 ^**c**^	76±16 ^**c**^	74±18 ^**c**^	60±20 ^**c**^	71±17	71±13 ^**c**^	73±10 ^**c**^	74±21 ^**c**^	76±20 ^**c**^	86±17 ^**c**^
**Social function**	81±18 ^**c**^	86±16 ^**c**^	86±14 ^**c**^	85±16 ^**c**^	77±18 ^**c**^	82±15	90±11 ^**c**^	82±9 ^**c**^	91±13 ^**c**^	86±19 ^**c**^	95±10 ^**c**^
**Academic function**	70±21 ^**c**^	74±18 ^**c**^	78±15 ^**c**^	71±16 ^**c**^	67±18 ^**c**^	73±12	85±15 ^**c**^	73±11 ^**c**^	75±20 ^**c**^	72±16 ^**c**^	83±17 ^**c**^
**Total score**	68±13 ^**c**^	80±13 ^**c**^	82±11 ^**c**^	80±12 ^**c**^	68±14 ^**c**^	77±11	82±12 ^**c**^	76±11 ^**c**^	80±4 ^**c**^	79±16 ^**c**^	88±11 ^**c**^
**Influencing factor of the paients total score**	Siblings in the family >2 Lipodystrophy HbA _1_c	NR	NR	Depression Adherence.HbA _1_c	NR	Age Sex HbA _1_c Hypoglycemia Hyperglycemia Onset of diabetes	NR	Duration of diabetes HbA _1_c Sex Age	HbA _1_c Age Sibling	Sex Sibling HbA _1_c Socioeconomic level Therapeutic education Hospitalization	Mothers’ and fathers’ educational status Fars’ occupation, Monitoring blood glucose

**CG**: control group. **HbA1c:** Glycosylated hemoglobin. **NR**: Not reported **. Par:** parents. **Pat:** patients. **Yr:** year.

Data were:

^a^ Minimum-maximum.

^b^ Mean.

^c^ Mean±standard deviation.

In this study, the patients’ total score was 68±13 ( Table 5). In the one hand, it was comparable to the one of the Iranian population of patients aged 8 to 18 years ^13^ who has a score of 68±14 ( Table 5). In the other hand, our score was lower than the ones reported in similar studies [ ***ie***, score ranging from 76±11 ^16^ to 88±11 ^8^]. In this study, while the patients’ social function score was high at 81±18, this of the emotional function was low at 53±20. Our results are intermediate with those reported in literature ( Table 5). First, almost all previous studies ^2^^,^^6^^,^^8^^,^^10^^–^^16^ reported that the social function score was the highest [ ***ie***; ranging from 77±18 ^13^ to 95±10 ^8^ ( Table 5)]. Second, while some previous studies, ^2^^,^^10^^,^^11^^,^^13^^–^^16^ reported that the emotional function score was the lowest [ ***ie***; ranging from 60±20 ^13^ to 76±16 ^11^ ( Table 5)], three studies ^6^^,^^12^^,^^13^ identified that the academic function score was the lowest [ ***ie***; ranging from 67±18 ^13^ to 72±16 ^6^ ( Table 5)].

The discrepancy between results could be explained partly by the age of included patients ranging from 2-18 years ^11^^,^^15^^,^^16^ to 8-18 years. ^2^^,^^6^^,^^13^ In addition, according to Abderrassoul et al., ^16^ impaired emotional function may be explained by the lack of autonomy and preoccupation with chronic complications. In order to increase the emotional function score, and to early detect and solve problems that children/adolescents may encounter, we recommend that HRQoL assessment after T1DM diagnosis should be a routine practice.

### Patients’ self-report vs. parents’ proxy-report of PedsQL4.0

The PedsQL4.0 scores of diabetic patients were comparable with those of their parents. This result is inconsistent with previous studies, which reported a significant difference between patients and their parents’ subjective perception of T1DM and the impact of the disease on their daily life. ^2^^,^^6^^,^^16^^,^^27^ The concordance between patient and parents results observed in this study can be explained by different factors, such as the presence of communication between parents and their children/adolescents, especially among children, who do not present generally a withdrawal and oppositional behavior before the pubertal period. ^2^^,^^14^ Moreover, parents seem to have more concern about their children/adolescents health when they reach school age, given the fact that kids haven’t yet developed an independent personality and weren’t became self-sufficient around this range of age.

### Influencing factors of the patients’ and parents’ PedsQL4.0 total scores

Table 5 exposes the influencing factor of the patients’ PedsQL4.0 scores among different populations of diabetic children/adolescents with TIDM. It appears that the following factors are independent predictors of HRQoL as perceived by diabetic patients: HbA _1_C, ^2^^,^^6^^,^^12^^,^^14^^,^^16^ age, ^2^^,^^14^^,^^16^ sex, ^6^^,^^14^^,^^16^ siblings, ^2^^,^^6^ depression, ^12^ adherence to treatment, ^12^ hypoglycemia, ^14^ hyperglycemia, ^14^ onset of T1DM, ^14^ duration of T1DM, ^16^ socioeconomic level, ^6^ therapeutic education, ^6^ hospitalization, ^6^ mothers’ and fathers’ educational status, ^8^ fathers’ occupation, ^8^ monitoring blood glucose. ^8^ In the present study, among all studies data, siblings in the family, lipodystrophy and HbA1C were independent predictors of HRQoL as perceived by diabetic patients, and lipodystrophy, siblings, BMI, hypoglycemia episodes, and HbA _1_C were independent predictors of HRQoL as perceived by parents ( Table 4). The following sentences will discuss the aforementioned factors.

An increase in the number of children in the family had a statistically significant effect on the HRQoL of children/adolescents as well as their parents ( Table 4). A number of “siblings higher than 2” reduces the patients’ HRQol total score by 6.84, and one unit of sibling reduced the parents’ HRQoL total score by 3.73 ( Table 4). Our results, which are in line with the findings of some studies from Turkey ^2^ and Ethiopia, ^6^ could be explained by the fact that increasing number of children in family may reduce parental support. Thus, siblings should be included in diabetes health education and diabetic children/adolescents should be supported by all family members. ^6^

Lipodystrophy was an independent predictor of HRQoL as perceived by both patients and parents ( Table 4). It appears that the presence of lipodystrophy reduces the patients and parents’ HRQoL total scores by 4.29 and 6.97, respectively. Besides, the changes of the skin and the unsightly aspect of lipodystrophy can also perturb the body image, especially among adolescents, and thus decreases the emotional function in HRQoL. To the best of the authors’ knowledge, no previous study has found an association between lipodystrophy and HRQoL ( Table 5).

HbA _1_C level (%) was an independent predictor of HRQoL as perceived by both patients and parents ( Table 4). One unit of HbA _1_C reduces the patients and parents’ HRQoL total scores by 1.20 and 0.96, respectively ( Table 4). Our result is in line with the findings of some previous studies reporting an association between lower HRQoL of patients and higher HbA _1_C levels. ^2^^,^^6^^,^^9^^,^^12^^,^^14^^,^^16^

BMI (kg/m ^2^) was an independent predictor of HRQoL as perceived by parents ( Table 4). In our study, we observed a noteworthy and positive correlation (r = 0.526), one unit of BMI increases the parents’ HRQoL total score by 1.03 ( Table 4). This finding aligns with two previous studies conducted in Saudi Arabia ^33^ and in Egypt. ^5^

Hypoglycemic episodes was an independent predictor of HRQoL as perceived by parents ( Table 4). Each hypoglycemic episode decreases the parents’ HRQoL total score by 1.69 ( Table 4). Our finding was consistent with other studies that indicated that treatment ongoing risk of hypoglycemia (especially nocturnal hypoglycemia) negatively affect the HRQOL of patients and their families. ^20^ In fact, hypoglycemia episodes are uncomfortable experiences and could cause an important psychological impact on the children/adolescents. Therefore, assessment of psychosocial burden, including fear of hypoglycemia, should be part of the management of T1DM. ^20^ Our findings suggest that motivating a child/adolescent to achieve optimal glycaemia levels and incorporating routine clinical assessment of HRQoL as an important component of diabetes management are necessary to determine the appropriate intervention to improve HRQoL. ^20^ Contrary to the present study, one Greece study ^14^ identified hypoglycemia as an independent predictor of HRQoL as perceived by diabetic patients ( Table 5).

Additional factors, such as age, ^2^^,^^14^^,^^16^ sex, ^6^^,^^14^^,^^16^ and socioeconomic data, ^6^^,^^8^ need to be discussed. First, in the present study, and similar to other studies, ^3^^,^^9^^,^^34^^–^^36^ there were no effects of age or sex on HRQoL. However, some previous studies reported that girls have lower total HRQoL scores than boys. ^6^^,^^9^^,^^15^^,^^16^^,^^18^^,^^37^ In general population, during adolescence, girls tend to have less self-esteem than boys, ^38^ and present more signs of depression, ^23^ which may constitute factors of a lower HRQoL. Moreover, adolescent girls with T1D are reported to be more anxious and less satisfied than boys, ^16^ and experience hormonal changes with an increase of insulin require. In our study, there was no significant association between socioeconomic status and patients’ HRQoL. This is consistent with the finding of a Norwegian study. ^36^ However, some other studies reported that a low socioeconomic level was significantly associated with sub-optimal management of T1DM, and this may negatively affect quality of life. ^6^^,^^8^^,^^9^^,^^39^

### Strengths and limitations of this study

The main two strengths of our study were the high enrolment rate (76%) and its pioneer character. Since it is the first study from the Great Maghreb, which assessed HRQoL and associated factors in a pediatric diabetic population.

Our study has three main limitations. The first limitation is related to the lack of a control group. The second limitation concerns the non-evaluation of some other potential determinant of HRQoL such as family and psychological relationships. The third limitation is related to the recruitment method from one center. Since all our patients were recruited from a single pediatric hospital, our results may not be easily generalized to children/adolescents with T1DM living in other locations in Tunisia or North Africa.

### Clinical implications

In clinical practice, providing support to patients with T1DM to guide them in reducing the risk of acute and chronic complications. Involving parents in the management of their children/adolescents’ disease and more particularly encouraging a constant commitment for the potential disease progression would eventually improve the HRQoL of children/adolescents as well as their parents. Therefore, our research emphasizes the significance of adequate management of diabetes for improved HRQoL, and conversely, the importance of good HRQoL for maintaining good diabetic control. To improve the low emotional and school function scores identified in the study, it is advisable to regularly evaluate the HRQoL of these children to detect any significant deterioration early on. This will facilitate the implementation of appropriate interventions that can enhance the overall management of the disease. Additionally, in families with multiple children/adolescents, it is essential to involve the siblings in diabetes education and empower them to take responsibility. Providing families with information on available social support resources can also be helpful.

## Conclusion

The study indicated that the PedsQL4.0 scores were satisfactory overall, with higher scores on the social function subscale, and lower scores in emotional and school functions. Furthermore, PedsQL4.0 scores for children/adolescents patients, and their parents were comparable. HRQoL of children/adolescents was influence significantly by sibling relationships, lipodystrophy, and HbA _1_C levels. Therefore, assessing the HRQoL after a diabetes diagnosis can aid in identifying and addressing potential challenges early on for children/adolescents.

## Ethical approval

The study’s ethical approval was obtained from the ethics committee of the faculty of medicine of Sousse, Tunisia *(Reference: CEFMS 61/2021, date: September 7, 2021).*

## Informed consent

Written informed consent was obtained from all parents of the children/adolescents after receiving an explanation of the study. In addition, adolescents over 13 years old signed the consent form

## Declaration

We have looked for assistance from artificial intelligence ( ***ie***; language model, ChatGPT 3.5) in the language editing our scientific paper. ^40^

## Data Availability

***Zenodo***: Excel data of the 116 Tunisian participants and their parents [Data set]. Zenodo. https://doi.org/10.5281/zenodo.10900075. ^41^ The project contains the following underlying data
- [Excel Data of the 116 participants and parents.xls] (Excel file including the numerical data of the 116 patients and their parents). Data are available under the terms of the Creative Commons Attribution 4.0 International license (CC-BY 4.0). Zenodo: Influencing factors of health-related quality-of-life perceived by both children/adolescents patients with type-1 diabetes mellitus and their parents: A North-African study The project contains the following underlying data:
- [ ***Appendix A*:** Copy of the PedsQl4 French questionnaire] (Applied questionnaire). https://doi.org/10.5281/zenodo.10900088. ^42^
- [ ***Appendix B***: Copy of the translated PedsQl4 Arabic questionnaire] (Translated questionnaire). https://doi.org/10.5281/zenodo.10900100. ^43^
- [ **Appendix C**: Copy of the translated PedsQl4 English questionnaire] (Translated questionnaire). https://doi.org/10.5281/zenodo.10900109. ^44^ Data are available under the terms of the Creative Commons Attribution 4.0 International license (CC-BY 4.0). ***Zenodo:*** STROBE checklist for ‘[Influencing factors of health-related quality-of-life perceived by both children/adolescents patients with type-1 diabetes mellitus and their parents: A North-African study]. https://doi.org/10.5281/zenodo.10900117. ^45^

## References

[1] Nwose EU Digban KA Anyasodor AE : Development of public health program for type 1 diabetes in a university community: preliminary evaluation of behavioural change wheel. *Acta Biomed.* 2017 Oct 23;88(3):281–8. Epub 20171023. eng. 10.23750/abm.v88i3.5803 29083332 PMC6142846

[2] Ozyazicioglu N Avdal EU Saglam H : A determination of the quality of life of children and adolescents with type 1 diabetes and their parents. *Int. J. Nurs. Sci.* 2017 Apr 10;4(2):94–8. Epub 20170204. eng. 10.1016/j.ijnss.2017.01.008 31406726 PMC6626105

[3] Grudziaz-Sekowska J Zamarlik M Sekowski K : Assessment of selected aspects of the quality of life of children with type 1 diabetes mellitus in Poland. *Int. J. Environ. Res. Public Health.* 2021 Feb 22;18(4). Epub 20210222. eng. 10.3390/ijerph18042107 33671503 PMC7926510

[4] WHO: World health organisation: WHO report - African region tops world in undiagnosed diabetes: WHO analysis.(Last visit: April 12, 2024). Reference Source

[5] AbdelMoez Ali B Abdelhakm AA Abdelhameed MA : Quality of life in children with type i diabetes mellitus (T1D) in Minia governorate: Relationship with Mood and family attitudes. *J. Diabetes Metab.* 2017 01/01;08((02). 10.4172/2155-6156.1000725

[6] Girma D Murugan R Wondossen K : Health-related quality of life and its associated factors in children and adolescents with type1 diabetes, Addis Ababa, Ethiopia. *Glob. Pediatr. Health.* 2021;8:2333794X211030879. Epub 20210708. eng. 10.1177/2333794X211030879 34291125 PMC8274109

[7] Al-Akour N Khader YS Shatnawi NJ : Quality of life and associated factors among Jordanian adolescents with type 1 diabetes mellitus. *J. Diabetes Complicat.* 2010 Jan-Feb;24(1):43–7. Epub 20090522. eng. 10.1016/j.jdiacomp.2008.12.011 19464929

[8] Bekele BT Demie TG Worku F : Health-related quality-of-life and associated factors among children and adolescents with type 1 diabetes mellitus: A cross-sectional study. *Pediatric Health Med. Ther.* 2022;13:243–56. Epub 20220622. eng. 10.2147/PHMT.S364454 35769766 PMC9234181

[9] Souza MA Freitas R Lima LS : Health-related quality of life of adolescents with type 1 diabetes mellitus. *Rev. Lat. Am. Enfermagem.* 2019;27:e3210. Epub 20191205. eng. 10.1590/1518-8345.2961.3210 31826155 PMC6896811

[10] Varni JW Burwinkle TM Jacobs JR : The PedsQL in type 1 and type 2 diabetes: reliability and validity of the Pediatric Quality of Life Inventory Generic Core Scales and type 1 Diabetes Module. *Diabetes Care.* 2003 Mar;26(3):631–7. Epub 2003/03/01. eng. 10.2337/diacare.26.3.631 12610013

[11] Emmanouilidou E Galli-Tsinopoulou A Karavatos A : Quality of life of children and adolescents with diabetes of Northern Greek origin. *Hippokratia.* 2008 Jul;12(3):168–75. Epub 2008/10/17. eng. 18923667 PMC2504400

[12] Nansel TR Weisberg-Benchell J Wysocki T : Steering committee of the family management of diabetes s. Quality of life in children with type 1 diabetes: a comparison of general and diabetes-specific measures and support for a unitary diabetes quality-of-life construct. *Diabet. Med.* 2008 Nov;25(11):1316–23. Epub 2008/12/03. eng. 10.1111/j.1464-5491.2008.02574.x 19046222 PMC2597420

[13] Jafari P Forouzandeh E Bagheri Z : Health related quality of life of Iranian children with type 1 diabetes: reliability and validity of the Persian version of the PedsQL Generic Core Scales and Diabetes Module. *Health Qual. Life Outcomes.* 2011 Nov 23;9:104. Epub 20111123. eng. 10.1186/1477-7525-9-104 , 22112006 PMC3280935

[14] Kalyva E Malakonaki E Eiser C : Health-related quality of life (HRQoL) of children with type 1 diabetes mellitus (T1DM): self and parental perceptions. *Pediatr. Diabetes.* 2011 Feb;12(1):34–40. Epub 2010/06/16. eng. 10.1111/j.1399-5448.2010.00653.x 20546163

[15] Abdul-Rasoul M AlOtaibi F AlMahdi M : Reliability and validity of the Arabic version of the PedsQL TM 3.0 diabetes module. *J. Diabetes Mellit.* 2012 01/01;02(03):301–307. 10.4236/jdm.2012.23047

[16] Abdul-Rasoul M AlOtaibi F Abdulla A : Quality of life of children and adolescents with type 1 diabetes in Kuwait. *Med. Princ. Pract.* 2013;22(4):379–84. Epub 20130215. eng. 10.1159/000347052 23428425 PMC5586761

[17] Erikson EH : *Childhood and society.* 2nd ed. Erikson-New York: Norton;1963.

[18] Hoey H Aanstoot HJ Chiarelli F : Good metabolic control is associated with better quality of life in 2,101 adolescents with type 1 diabetes. *Diabetes Care.* 2001 Nov;24(11):1923–8. Epub 2001/10/27. eng. 10.2337/diacare.24.11.1923 11679458

[19] Reid AM Balkhi AM St. Amant J : Relations between quality of life, family factors, adherence, and glycemic control in pediatric patients with type 1 diabetes mellitus. *Child. Health Care.* 2013 2013/10/01;42(4):295–310. 10.1080/02739615.2013.842455

[20] Caferoglu Z Inanc N Hatipoglu N Kurtoglu S : Health-related quality of life and metabolic control in children and adolescents with type 1 diabetes mellitus. *J. Clin. Res. Pediatr. Endocrinol.* 2016 Mar 5;8(1):67–73. Epub 20151218. eng. 10.4274/jcrpe.2051 26758371 PMC4805051

[21] Sengbusch S von Muller-Godeffroy E Hager S : Mobile diabetes education and care: intervention for children and young people with Type 1 diabetes in rural areas of northern Germany. *Diabet. Med.* 2006 Feb;23(2):122–7. eng. 10.1111/j.1464-5491.2005.01754.x 16433708

[22] Bas V Bideci A Soysal Acar A : Evaluation of factors affecting quality of life in children with type 1 diabetes mellitus. *J. Diabetes Metab.* 2011;02(8):154. 10.4172/2155-6156.1000154

[23] AlBuhairan F Nasim M Al Otaibi A : Health related quality of life and family impact of type 1 diabetes among adolescents in Saudi Arabia. *Diabetes Res. Clin. Pract.* 2016 Apr;114:173–9. Epub 20160108. eng. 10.1016/j.diabres.2016.01.001 26830857

[24] Wagner J Heapy A James A : Brief report: glycemic control, quality of life, and school experiences among students with diabetes. *J. Pediatr. Psychol.* 2006 Sep;31(8):764–9. Epub 20050914. eng. 10.1093/jpepsy/jsj082 16162839

[25] Elm E von Altman DG Egger M : The strengthening the reporting of observational studies in epidemiology (STROBE) statement: guidelines for reporting observational studies. *Int. J. Surg.* 2014 Dec;12(12):1495–9. Epub 20140718. 10.1016/j.ijsu.2014.07.013 25046131

[26] Kang M Ragan BG Park JH : Issues in outcomes research: an overview of randomization techniques for clinical trials. *J. Athl. Train.* 2008 Apr-Jun 43(2):215–21. Epub 2008/03/18. eng. 18345348 10.4085/1062-6050-43.2.215 PMC2267325

[27] Abolfotouh MA Kamal MM El-Bourgy MD : Quality of life and glycemic control in adolescents with type 1 diabetes and the impact of an education intervention. *Int. J. Gen. Med.* 2011 Feb 20;4:141–52. Epub 20110220. eng. 10.2147/IJGM.S16951 21475630 PMC3068879

[28] Ingersoll GM Marrero DG : A modified quality-of-life measure for youths: psychometric properties. *Diabetes Educ.* 1991 Mar-Apr;17(2):114–8. Epub 1991/03/01. eng. 10.1177/014572179101700219 1995281

[29] Cole TJ Bellizzi MC Flegal KM : Establishing a standard definition for child overweight and obesity worldwide: international survey. *BMJ (Clinical research ed).* 2000 May 6;320(7244):1240–3. Epub 2000/05/08. eng. 10.1136/bmj.320.7244.1240 10797032 PMC27365

[30] Ben Saad H Prefaut C Missaoui R : Reference equation for 6-min walk distance in healthy North African children 6-16 years old. *Pediatr. Pulmonol.* 2009 Apr;44(4):316–324. 10.1002/ppul.20942 19330774

[31] Varni JW Seid M Kurtin PS : PedsQL 4.0: reliability and validity of the pediatric quality of life inventory version 4.0 generic core scales in healthy and patient populations. *Med. Care.* 2001 Aug;39(8):800–12. Epub 2001/07/27. eng. 10.1097/00005650-200108000-00006 11468499

[32] Graue M Wentzel-Larsen T Bru E : The coping styles of adolescents with type 1 diabetes are associated with degree of metabolic control. *Diabetes Care.* 2004 Jun;27(6):1313–7. Epub 2004/05/27. eng. 10.2337/diacare.27.6.1313 15161781

[33] Al-Hayek AA Robert AA Abbas HM : Assessment of health-related quality of life among adolescents with type 1 diabetes mellitus in Saudi Arabia. *Saudi Med. J.* 2014 Jul;35(7):712–7. Epub 2014/07/17. eng. 25028228

[34] Murillo M Bel J Perez J : Health-related quality of life (HRQOL) and its associated factors in children with Type 1 Diabetes Mellitus (T1DM). *BMC Pediatr.* 2017 Jan 13;17(1):16. Epub 2017/01/13. 10.1186/s12887-017-0788-x 28086765 PMC5237211

[35] Laffel LM Connell A Vangsness L : General quality of life in youth with type 1 diabetes: relationship to patient management and diabetes-specific family conflict. *Diabetes Care.* 2003 Nov;26(11):3067–73. Epub 2003/10/28. eng. 10.2337/diacare.26.11.3067 14578241

[36] Froisland DH Graue M Markestad T : Health-related quality of life among Norwegian children and adolescents with type 1 diabetes on intensive insulin treatment: a population-based study. *Acta Paediatr.* 2013 Sep;102(9):889–95. Epub 20130716. eng. 10.1111/apa.12312 23738648

[37] Babiker A Al Aqeel B Marie S : Quality of Life and glycemic control in Saudi children with type 1 diabetes at different developmental age groups. *Clin. Med. Insights Endocrinol. Diabetes.* 2021;14:1179551421990678. Epub 20210211. eng. 33628072 10.1177/1179551421990678 PMC7883141

[38] Rowe ML : Understanding socioeconomic differences in parents’ speech to children. *Child Dev. Perspect.* 2017 2018/06/01;12(2):122–7. 10.1111/cdep.12271

[39] Hassan K Loar R Anderson BJ : The role of socioeconomic status, depression, quality of life, and glycemic control in type 1 diabetes mellitus. *J. Pediatr.* 2006 Oct;149(4):526–31. Epub 2006/10/03. eng. 10.1016/j.jpeds.2006.05.039 17011326

[40] Dergaa I Zakhama L Dziri C : Enhancing scholarly discourse in the age of artificial intelligence: A guided approach to effective peer review process. *Tunis. Med.* 2023 Oct 5;101(10):721–6. Epub 20231005. eng. 38465750 PMC11261523

[41] Ben Saad H Ben AI : Excel data of the 116 Tunisian participants and their parents.[Data set]. *Zenodo.* 2024. (Last visit: April 12, 2024). 10.5281/zenodo.10900075

[42] Ben Saad H Ben AI : Copy of the PedsQl4 French questionnaire. *Zenodo.* 2024. (Last visit: April 12, 2024). 10.5281/zenodo.10900088

[43] Ben Saad H Ben AI : Copy of the translated PedsQl4 Arabic questionnaire. *Zenodo.* 2024. (Last visit: April 12, 2024). 10.5281/zenodo.10900100

[44] Ben Saad H Ben AI : Copy of the translated PedsQl4 English questionnaire. *Zenodo.* 2024. (Last visit: April 12, 2024). 10.5281/zenodo.10900109

[45] Ben Saad H Ben AI : STROBE checklist for the original study titled “Influencing factors of health-related quality-of-life perceived by both children/adolescents patients with type-1 diabetes mellitus and their parents: A North-African study”. *Zenodo.* 2024. (Last visit: April 12, 2024). 10.5281/zenodo.10900117 PMC11795355 39911881

