# Supplementary material for: Influencing factors of health-related quality-of-life perceived by both children/adolescents patients with type-1 diabetes mellitus and their parents: A North-African study
Source: F1000Res. 2025 Jan 17;13:429. Originally published 2024 Apr 30. [Version 2] doi: 10.12688/f1000research.148074.2 (PMC11795355; doi:10.12688/f1000research.148074.2)
Supplement: Peer review history

# Reviewer report: Reviewer response for version 1

**DOI:** 10.5256/f1000research.162341.r277118

**Published:** 2024-12-19

**Authors:** Ihsen Zairi

**Affiliations:** 1 Faculté de Médecine de Tunis, Université de Tunis El Manar, Tunis, Tunisia

It’s an interesting article, the author  determine the influencing factors of health-related quality-of-life (HRQoL) perceived by North-African children/adolescents with type-1 diabetes-mellitus (T1DM) and their parents.

  «  The aim of this study was To determine the influencing factors of health-related quality-of-life (HRQoL) perceived by North-African children/adolescents with type-1 diabetes-mellitus (T1DM) and their parents »

 The methodology section is detailed, the author explains data collection and statistical analysis very well

 The  “result section” meets the objective of the article and the results are well presented in the text and tables

 The  “discussion section” the results of the article are well discussed with the literature and the bibliography is recent.

 Major points are  :

^-                      ^ the subject because Type 1 diabetes mellitus (T1DM) is a major public health problem in north Africa,  

^-                      ^the study design, It was a cross-sectional study 

^-                      ^the sample size was appraised according to the following formula ^26^: *N* = ( *Z* *_α_*) ^2^ *s* ^2^/ *d* ^2^, where “ ***s***” is the standard deviation (SD= 9.79) and “ ***d***” is the accuracy of estimate or how close it is to the true mean (= 76.51). Given the pioneering nature of this study.

 -                   sufficient details of methods and analysis are provided to allow replication by others

^-                      ^the work clearly and accurately presented and  it cite the current literature

^-                      ^the statistical analysis and its interpretation are appropriate

^-                      ^all the source data underlying the results are available to ensure full reproducibility

^-                      ^the conclusions drawn are adequately supported by the results

Is the work clearly and accurately presented and does it cite the current literature?

Yes

If applicable, is the statistical analysis and its interpretation appropriate?

Yes

Are all the source data underlying the results available to ensure full reproducibility?

Yes

Is the study design appropriate and is the work technically sound?

Yes

Are the conclusions drawn adequately supported by the results?

Yes

Are sufficient details of methods and analysis provided to allow replication by others?

Yes

Reviewer Expertise:

pediatric cardiology

I confirm that I have read this submission and believe that I have an appropriate level of expertise to confirm that it is of an acceptable scientific standard.

# Reviewer report: Reviewer response for version 1

**DOI:** 10.5256/f1000research.162341.r277117

**Published:** 2024-12-17

**Authors:** Sameh Mabrouk

**Affiliations:** 1 Pediatrics Department, Sahloul University Hospital, Sousse, Tunisia

This article about “Influencing factors of health-related quality-of-life perceived by both children/adolescents patients with type-1 diabetes mellitus and their parents: A North-African study” by Ms Ben Abdesselem, is an original article that accurately evaluates the quality of life of children/adolescents with type diabetes as well as their parents with a particular focus on influencing factors of HRQL. It is it is an unprecedented study in Tunisian as well as north African pediatric settings.

 This article accurately evaluates the quality of life of children/adolescents with type I diabetes as well as their parents. And focuses on influencing factors of HRQL.

 It is an unprecedented study in the Tunisian pediatric setting.

 The introduction is too long and should be shortened. Some of it's parts can be withdrawn.

 The study design was appropriate to the study objectives, and explained with details, making it reproductible if needed.

 Results were well represented with tables, and accurately interpreted . The results responded to the author's objectives.

 The discussion part was thorough, with authors' self-criticism. The results were discussed according to literature data on the subject.

 References were mostly recent.

 Globally it is an interesting and good study. The authors, methodically addressed a subject of actuality, in the era of increasing incidence of type I diabetes among children, and integrative medicine focusing on quality of life as a major part of chronic disease management.

Is the work clearly and accurately presented and does it cite the current literature?

Yes

If applicable, is the statistical analysis and its interpretation appropriate?

Yes

Are all the source data underlying the results available to ensure full reproducibility?

Yes

Is the study design appropriate and is the work technically sound?

Yes

Are the conclusions drawn adequately supported by the results?

Yes

Are sufficient details of methods and analysis provided to allow replication by others?

Yes

Reviewer Expertise:

pediatric nephrology

I confirm that I have read this submission and believe that I have an appropriate level of expertise to confirm that it is of an acceptable scientific standard.

# Response

**Published:** 2025-01-06

**Authors:** IMEN BEN ABDESSELEM

**Affiliations:** Pediatrics, Hospital Farhat Hached Sousse, Sousse, Tunisia

The authors sincerely thank the reviewer for her thorough and insightful feedback on our manuscript. We have carefully considered each comment and made the necessary revisions to address his concerns. We believe these changes have significantly improved the manuscript. Below are our specific responses to the reviewer positive comments/suggestions.

**COMMENT 1**. I. General comment:

 It’s an interesting article, the author determine the influencing factors of health-related quality-of-life (HRQoL) perceived by North-African children/adolescents with type-1 diabetes-mellitus (T1DM) and their parents. «The aim of this study was To determine the influencing factors of health-related quality-of-life (HRQoL) perceived by North-African children/adolescents with type-1 diabetes-mellitus (T1DM) and their parents».

**RESPONSE**

 We thank the reviewer for his valuable feedback.

**COMMENT 2**.

 The methodology section is detailed, the author explains data collection and statistical analysis very well

**RESPONSE**

 We thank the reviewer for his valuable feedback.

**COMMENT 3**.

 The “result section” meets the objective of the article and the results are well presented in the text and tables. The “discussion section” the results of the article are well discussed with the literature and the bibliography is recent.

**RESPONSE**

 We thank the reviewer for his valuable feedback

**COMMENT 4**.

 Major points are:

- The subject because Type 1 diabetes mellitus (T1DM) is a major public health problem in north Africa,
- The study design, It was a cross-sectional study 
- The sample size was appraised according to the following formula 26: N = (Zα)2 s2/d2, where “s” is the standard deviation (SD= 9.79) and “d” is the accuracy of estimate or how close it is to the true mean (= 76.51). Given the pioneering nature of this study.
- Sufficient details of methods and analysis are provided to allow replication by others
- The work clearly and accurately presented and  it cite the current literature
- the statistical analysis and its interpretation are appropriate
- All the source data underlying the results are available to ensure full reproducibility
- The conclusions drawn are adequately supported by the results

**RESPONSE**

 We thank the reviewer for his valuable feedback.
